# Assessment of CDASI scoring by a multimodal large language model: a comparative study with expert assessors

**DOI:** 10.1007/s00296-026-06205-1

**Published:** 2026-07-08

**Authors:** Marco Fornaro, Vincenzo Venerito, Swapnasha Panigrahi, Sara Sabbagh, Florenzo Iannone, Latika Gupta

**Affiliations:** 1 Unit of Rheumatology, Department of Precision and Regenerative Medicine, University of Bari, Area Jonica (DiMePRe-J), Bari, Italy; 2 University of Birmingham, Birmingham, UK; 3 Division of Rheumatology, Department of Pediatrics, Medical College of Wisconsin, Milwaukee, USA; 4 Department of Rheumatology, Royal Wolverhampton Hospitals NHS Trust, Wolverhampton, UK; 5 Department of Infection, School of Infection, Infammation and Immunology, College of Medicine and Health, University of Birmingham, Birmingham, UK; 6 Francis Crick Institute, London, UK

**Keywords:** Dermatomyositis, Artificial intelligence, Large language models, Disease activity, Skin diseases

## Abstract

Large language models (LLMs) with multimodal capabilities may support automated assessment of cutaneous disease activity in dermatomyositis (DM). We evaluated the performance of Claude v3.5 Sonnet in scoring the Cutaneous Dermatomyositis Disease Area and Severity Index (CDASI) compared with expert rheumatologists. Thirty published DM cases with available clinical images were retrospectively analyzed. Two expert rheumatologists independently scored CDASI activity and damage domains. Claude v3.5 Sonnet assessed the same images using structured prompting based on CDASI definitions. Agreement was evaluated using intraclass correlation coefficients (ICCs) with a two-way random-effects model for absolute agreement. The LLM demonstrated better agreement for activity than for damage assessment. Global CDASI activity showed good agreement with expert raters (ICC 0.71, 95% CI 0.45–0.86), whereas damage reliability was lower (ICC 0.41, 95% CI 0.25–0.67). Agreement was moderate for activity-related domains including erythema (ICC 0.61), scaling (ICC 0.57), and erosions (ICC 0.57), while lower concordance was observed for chronic damage-related features such as poikiloderma (ICC 0.47, 95% CI 0.10–0.73). Hand assessments demonstrated the strongest performance, particularly for periungual changes (ICC 1.0, 95% CI 1.0–1.0) and global hand scores (ICC 0.95, 95% CI 0.91–0.97). The model also markedly reduced evaluation time compared with clinicians (42 s vs. 8.4 min per case), corresponding to an approximately 92% reduction in scoring time. Multimodal LLMs demonstrated promising agreement with expert raters for CDASI activity assessment and markedly improved efficiency, although lower reliability in damage domains reinforces the continued need for physician oversight.

**Supplementary Information:**

The online version contains supplementary material available at 10.1007/s00296-026-06205-1.

## Introduction

Dermatomyositis (DM) is a rare idiopathic inflammatory myopathy characterized by distinctive cutaneous manifestations that may persist independently of muscle involvement [1]. Accurate and reproducible assessment of skin disease is essential for clinical monitoring, therapeutic decision-making, and clinical trial enrollment [2]. The Cutaneous Dermatomyositis Disease Area and Severity Index (CDASI) is the most widely validated instrument for quantifying disease activity and damage, with demonstrated reliability and responsiveness in both observational and interventional settings [3]. However, CDASI scoring is time-consuming and requires trained assessors, limiting its widespread adoption in routine practice and multicenter studies.

In parallel, artificial intelligence has shown increasing promise in medical domains requiring pattern recognition, classification, and structured decision-making [4–7]. Large language models (LLMs) trained on massive text and image corpora are now capable of performing tasks that traditionally required human clinical expertise, including diagnostic interpretation and structured data abstraction [8]. While initial studies have demonstrated encouraging results in applying LLMs to disease activity tools such as the Myositis Disease Activity Assessment Tool (MDAAT) [9], evidence regarding their ability to assess dermatologic features remains limited; however, promising results are emerging from other series [10].

We therefore evaluated the performance of a state-of-the-art LLM (Claude v3.5 Sonnet) in scoring cutaneous manifestations of DM using CDASI criteria. We compared its outputs with those of expert rheumatologists and assessed agreement across global and domain-specific scores, with particular focus on differences between activity and damage components.

## Materials and methods

We conducted a retrospective analysis of 30 DM cases identified through a systematic PubMed search (Supplementary Fig. 1). Each case included high-quality clinical images suitable for CDASI scoring across different anatomical regions and disease severities.

Two expert rheumatologists (M.F. and S.S.), both experienced in myositis assessment and CDASI scoring, independently assessed all images using standardized CDASI definitions, blinded to each other’s scores and to model outputs. Both raters are involved as principal or sub-investigators in at least two myositis clinical trials and routinely manage outpatient clinics including more than 50 patients with DM. Scoring included both CDASI global activity and CDASI global damage, as well as individual domains (erythema, scaling, erosions/ulceration, poikiloderma, calcinosis, and hand-specific features), according to the anatomical areas depicted in the images.

**Fig. 1:**
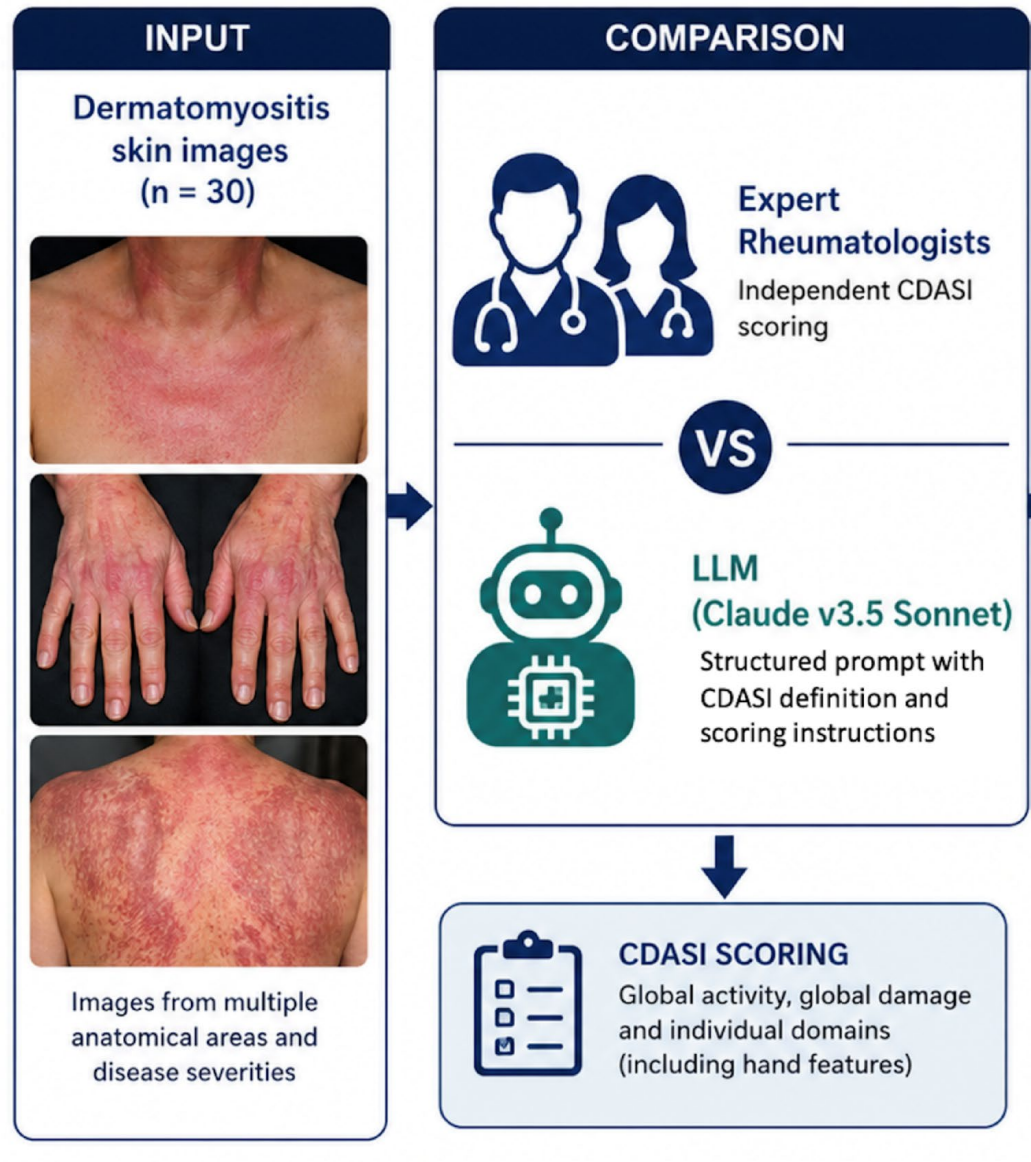
Study workflow comparing expert rheumatologists and a multimodal large language model for CDASI scoring in dermatomyositis

The LLM (Claude v3.5 Sonnet) was provided with the same images and prompted using a structured chain-of-thought approach incorporating detailed CDASI definitions and scoring instructions. Prompting followed a structured, chain-of-thought design built on the validated modified CDASI [3]. The model was assigned the role of an assessor experienced in the cutaneous evaluation of DM and instructed to score only the visible skin, without offering a diagnosis or management advice. The operational CDASI definitions were supplied verbatim (supplementary material), separating activity components, scored per anatomical area (erythema 0–3: absent, pink, red, dark red/violaceous; scale 0–2: absent, superficial, thick/crusted; erosion/ulceration 0–2: absent, erosion, ulceration), together with Gottron’s papules/sign, periungual changes and scalp alopecia, from damage components scored per area (poikiloderma and calcinosis, each absent/present). The model was required to reason about each component before assigning a score and to return the scores in a fixed structure that was then mapped onto the CDASI global activity score, the CDASI global damage score and the individual domains, including hand-specific features. The model was accessed through the standard Claude interface with default parameters; no fine-tuning, retrieval augmentation, few-shot exemplars, or dermatology-specific training data were used. Outputs were mapped to CDASI scoring categories to ensure consistency with expert assessments (Fig. 1).

Agreement between raters was evaluated using intraclass correlation coefficients (ICCs) calculated with a two-way random-effects model for absolute agreement. ICCs were computed for global activity, global damage, and individual domains. 95% confidence intervals (95% CI) were reported. Statistical analyses were performed using Stata version 18. This study was based exclusively on previously published, anonymized clinical images retrieved from the literature and did not involve direct patient participation or access to identifiable patient data; therefore, formal ethics committee approval and informed consent were not required according to local regulations. The GAMER checklist for generative AI has been included as supplementary Table 1.

## Results

Agreement between the LLM and expert assessors varied across domains, with higher reliability observed for activity-related features than for damage (Table 1).

**Table 1:** Intraclass Correlation Coefficient of CDASI in 30 Dermatomyositis cases

Domain	ICC	95% CI
CDASI global activity	0.71	0.45–0.86
CDASI global damage	0.41	0.25–0.67
Erythema	0.61	0.27–0.81
Scaling	0.57	0.19–0.79
Erosions	0.57	0.21–0.79
Poikiloderma	0.47	0.10–0.73
Calcinosis	Not Detected	N/A
Hands (Global)	0.95	0.91–0.97
Hands (Erythema)	0.78	0.24–0.95
Hands (Ulcers)	0.65	0.10–0.92
Hands (Damage)	0.37	0.10–0.85
Hands (Periungual changes)	1.0	1.0–1.0

CI: confidence interval; ICC: Intraclass Correlation Coefficient

For CDASI global activity, agreement was good (ICC 0.71, 95% CI 0.45–0.86). In contrast, CDASI global damage demonstrated lower reliability (ICC 0.41, 95% CI 0.25–0.67).

At the domain level, agreement was moderate for erythema (ICC 0.61), scaling (0.57), and erosions (0.57), indicating consistent performance in identifying features of active inflammation. Agreement was lower for poikiloderma (ICC 0.47), reflecting greater variability in interpreting chronic or mixed lesions. Calcinosis was not detected in any case by either the LLM or expert raters.

Hand assessments showed the highest levels of agreement. The global hand score demonstrated excellent reliability (ICC 0.95, 95% CI 0.91–0.97). Agreement was strong for hand erythema (ICC 0.78) and moderate for hand ulcers (ICC 0.65), while concordance for hand damage was lower (ICC 0.37). Periungual changes showed near-perfect agreement (ICC 1.0), likely reflecting the distinct visual characteristics of these features.

Beyond accuracy, efficiency emerged as a major advantage of LLM use. Claude completed scoring for each case in an average of 42 s (range 35–50 s), in contrast to the 8.4 min per case (range 6–12 min) required by expert clinicians, corresponding to a 92% reduction in evaluation time.

## Discussion

In this study, a multimodal LLM demonstrated good concordance with expert rheumatologists in assessing CDASI global activity, while performance was lower for global damage and domains reflecting chronic cutaneous changes. Compared with previous reports on the use of LLMs in rheumatology, this study provides preliminary evidence on image-based CDASI scoring in DM, demonstrating stronger performance for activity-related features than for damage domains and emphasizing the need for physician-supervised interpretation. These findings align with known characteristics of the CDASI, in which activity measures are generally more reproducible than damage assessments [11].

The model performed best in domains with clear and well-defined visual features, such as erythema and periungual changes, and in anatomically standardized regions such as the hands. In contrast, lower agreement in poikiloderma and damage domains highlights the difficulty of interpreting subtle, heterogeneous, or chronic lesions—challenges that are also observed among human raters.

The discrepancy between activity and damage likely reflects intrinsic limitations of current LLMs [12, 13]. While these models can effectively recognize visually salient inflammatory features, they lack the contextual clinical judgment required to interpret long-standing or structurally complex changes [14]. This limitation is particularly relevant for longitudinal disease assessment and outcome evaluation.

From a clinical research perspective, the ability of LLMs to provide reproducible activity scoring may offer practical advantages, particularly in multicenter trials where standardization and efficiency are critical [15, 16]. Automated or semi-automated scoring could support patient pre-screening and reduce inter-rater variability. However, the lower reliability observed for damage underscores the need for continued human oversight.

Several limitations should be acknowledged. The relatively small sample size limits statistical precision and generalizability; therefore, this study should be considered exploratory and hypothesis-generating. The dataset was based on previously published clinical images selected for privacy reasons, which are generally of higher quality than routine clinical photographs and may introduce selection bias; therefore, generalizability to lower-resolution real-world images remains to be established. Model performance also depended on image quality and structured prompting, and the LLM was not specifically trained on dermatologic datasets. In addition, scoring was performed once per image using default model settings, without assessment of intra-model reproducibility across repeated runs or prompt variations. Finally, Claude v3.5 Sonnet was selected because it represented one of the most advanced multimodal LLMs available at the time the study was conducted; however, the rapid evolution of AI models may influence reproducibility and comparative performance over time. Future studies should evaluate performance in larger prospective cohorts and real-world clinical settings.

In conclusion, Claude v3.5 Sonnet demonstrated encouraging agreement with expert raters for CDASI activity assessment, although reliability was lower in damage-related domains. These exploratory and hypothesis-generating findings suggest that multimodal LLMs may have potential as adjunctive research-support tools for standardized disease activity assessment, while reinforcing the continued need for physician oversight and integrated human–AI evaluation approaches.

## Supplementary Information

Below is the link to the electronic supplementary material.

Supplementary Material 1

Supplementary Material 2

## Data Availability

Data will be shared by the corresponding author upon request.

## Declarations

### Artificial Intelligence-Assisted Technology Statement

ChatGPT (OpenAI, GPT-5-based model) was used for language editing and manuscript refinement, while Claude 3.5 Sonnet (Anthropic) was evaluated as the study tool for scoring cutaneous dermatomyositis activity from clinical images. All AI-generated outputs were critically reviewed, verified, and edited by the authors, who take full responsibility for the accuracy, integrity, and originality of the manuscript. No AI-assisted technology was listed as an author or co-author.
