# Supplementary material for: Assessment of CDASI scoring by a multimodal large language model: a comparative study with expert assessors
Source: Rheumatol Int. 2026 Jul 8;46(7):196. doi: 10.1007/s00296-026-06205-1 (PMC13346326; doi:10.1007/s00296-026-06205-1)
Supplement: Supplementary file 1 — Supplementary Material 1 [file 296_2026_6205_MOESM1_ESM.docx]

**Supplementary Table 1.** GAMER checklist

| **No.** | **Item** | **Response** |
| --- | --- | --- |
| 1 | Did you use any Generative Artificial Intelligence (GAI) tools (such as large language models or large visual models) in any section or step of this manuscript or study? | Yes |
| 2 | Specify the GAI tool(s) used, their versions and/or release dates and the date(s)/period the tools were used. | ChatGPT (OpenAI, GPT-5-based model) and Claude 3.5 Sonnet (Anthropic) were used in this study. ChatGPT was used for language editing and manuscript refinement, while Claude 3.5 Sonnet was evaluated as the study tool for scoring cutaneous dermatomyositis activity from clinical images in comparison with expert human assessment. All AI-generated outputs were critically reviewed, verified, and approved by the authors. |
| 3 | Describe whether a specific prompting technique was used to generate any content of the manuscript or to perform analyses during the study. Please also provide the unedited responses to the prompts. | A specific prompting technique was used for the study analysis but not for manuscript text generation. Claude 3.5 Sonnet (Anthropic) received a single structured, chain-of-thought scoring instruction built on the validated modified CDASI (full template above), combining explicit role assignment, the verbatim CDASI operational definitions for activity and damage, a stepwise reasoning protocol, and a fixed reporting structure mapped to the CDASI global activity, global damage and individual domains. No prompting technique was used to generate the scientific content of the manuscript; ChatGPT (OpenAI, GPT-5-based model) was used only for language editing of author-written text. The unedited per-case model responses are available from the corresponding author upon reasonable request.  ROLE You are an expert assessor of cutaneous dermatomyositis, applying the validated modified Cutaneous Dermatomyositis Disease Area and Severity Index (CDASI).  TASK You will be shown clinical photographs of patients with dermatomyositis. For each image, score only the skin involvement that is visible, using the CDASI. Do not provide a diagnosis or management advice; do not infer findings that are not visible.  CDASI DEFINITIONS ACTIVITY - scored for EACH visible anatomical area:  - Erythema (0-3): 0 absent; 1 pink; 2 red; 3 dark red / violaceous (dusky)  - Scale (0-2): 0 absent; 1 superficial; 2 thick / crusted  - Erosion/ulceration (0-2): 0 absent; 1 erosion; 2 ulceration Additional ACTIVITY items, when visible:  - Gottron's papules / Gottron's sign  - Periungual changes (nailfold capillary changes / periungual erythema)  - Scalp alopecia Global activity score: sum across areas and items (range 0-100).  DAMAGE - scored for EACH visible anatomical area (absent = 0 / present = 1):  - Poikiloderma (telangiectasia + dyspigmentation)  - Calcinosis Global damage score: sum across areas (range 0-32).  REASONING PROTOCOL (think step by step) 1. Describe the image and identify which anatomical area(s) are visible. 2. For each visible area, reason about each activity component before scoring it. 3. If the hands are visible, assess Gottron's papules/sign, hand erythema, hand  ulcers and periungual changes. 4. Assess damage (poikiloderma, calcinosis) for each visible area. 5. Map the findings to CDASI categories and report:  - per-area component scores with a brief justification  - CDASI global activity (visible)  - CDASI global damage (visible) |
| 4 | If a new GAI tool was developed or fine-tuned based on an existing AI model, report the name and version of the original model. | N/A |
| 5 | Describe the role of GAI tools in all phases of this study where they were used (including manuscript writing). | GAI tools were used to support manuscript preparation. ChatGPT contributed to language editing and readability improvement, whereas Claude 3.5 Sonnet assisted in the evaluation of skin involvement scores from clinical images. |
| 6 | Report the specific section or paragraphs of the manuscript that GAI tools contributed to. | GAI tools contributed to language refinement across multiple manuscript sections, including the Introduction, Results, Discussion and Abstract, and assisted in the assessment of skin involvement scores from clinical images. |
| 7 | Describe how the content generated by GAI tools was verified and (when necessary) modified. | All AI-assisted content were critically reviewed and verified by the authors to ensure consistency with the original data, scientific accuracy and appropriateness of interpretation. |
| 8 | Describe how data privacy and confidentiality were ensured during the use of GAI tools. | No directly identifiable patient data or confidential information were entered into the GAI tools. Only anonymised clinical images and aggregated data were used. |
| 9 | Describe whether and how the use of GAI tools may have influenced the interpretation of results, the study’s overall accuracy, or conclusions. | GAI tools did not perform statistical analyses or determine study conclusions. Final interpretation of results and scientific conclusions remained entirely under the responsibility of the authors. |
