# Supplementary material for: Assessment of CDASI scoring by a multimodal large language model: a comparative study with expert assessors
Source: Rheumatol Int. 2026 Jul 8;46(7):196. doi: 10.1007/s00296-026-06205-1 (PMC13346326; doi:10.1007/s00296-026-06205-1)
Supplement: Supplementary file 2 — Supplementary Material 2 [file 296_2026_6205_MOESM2_ESM.docx]

**Supplementary figure 1.** Case Series and Scoring Details Supporting LLM-CDASI Analysis

Case 1: Bennett, R.; Bradley, K.; Salem, I.; Weiner, D.; Patel, D.; Cloutier, J.; Pace, N.; Barton, D. A Case of Paraneoplastic Anti-TIF1-γ Antibody-Positive Dermatomyositis Presenting with Generalized Edema and Associated with Aortic Aneurysm. *Dermato* **2023**, *3*, 232-240. https://doi.org/10.3390/dermato3040018
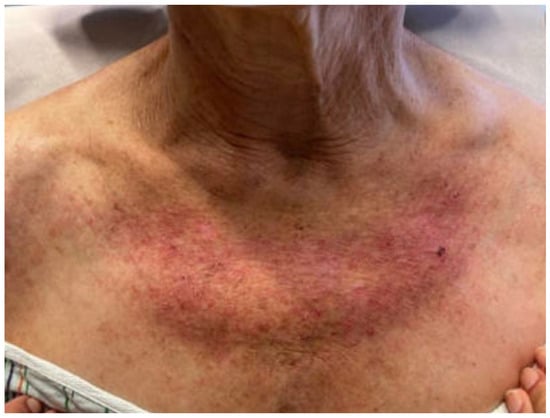


Score V-area neck (frontal)

Case 2: Bennett, R.; Bradley, K.; Salem, I.; Weiner, D.; Patel, D.; Cloutier, J.; Pace, N.; Barton, D. A Case of Paraneoplastic Anti-TIF1-γ Antibody-Positive Dermatomyositis Presenting with Generalized Edema and Associated with Aortic Aneurysm. *Dermato* **2023**, *3*, 232-240. https://doi.org/10.3390/dermato3040018


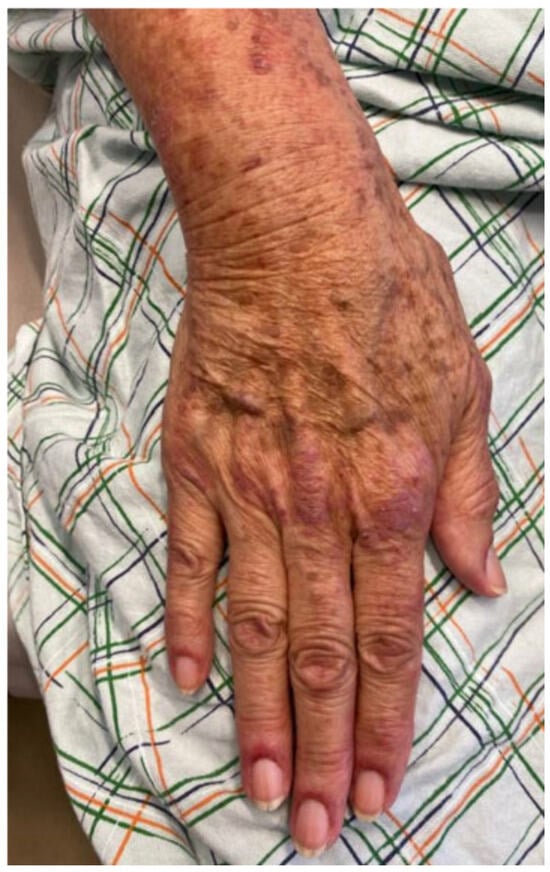


Score hand

Case 3: Grinnell M, Keyes E, Vazquez T, Concha J, Diaz D, Wat M, Elenitsas R, Werth VP. Dermatomyositis associated with hyponatremia and anasarca. JAAD Case Rep. 2021 Aug 19;16:86-89. doi: 10.1016/j.jdcr.2021.08.007. PMID: 34541273; PMCID: PMC8435987.
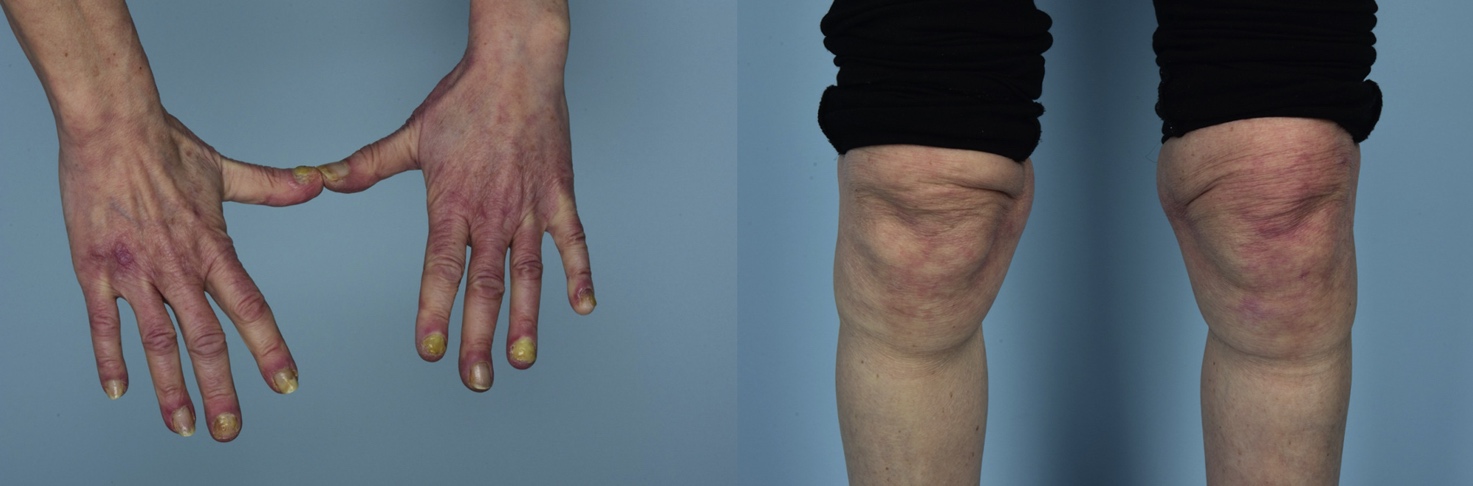


Score Gottron’s not on hands

Case 4: Feldman BM, Rider LG, Reed AM, Pachman LM. Juvenile dermatomyositis and other idiopathic inflammatory myopathies of childhood. Lancet. 2008 Jun 28;371(9631):2201-12. doi: 10.1016/S0140-6736(08)60955-1. PMID: 18586175.
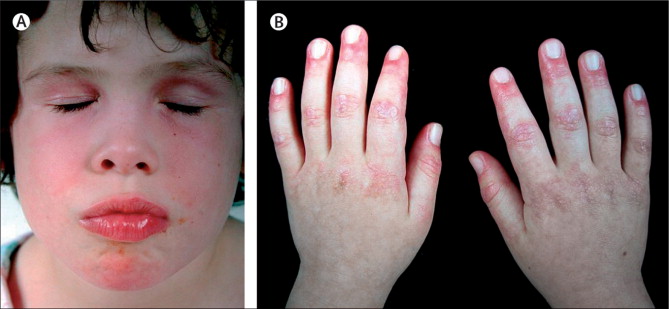


Score periorbital and hands

Case 5: Salgueiro C, Poblete MJ, Robles-Silva C, Abarzúa Á, Vera-Kellet C. Trichoscopic, oral, and periungual fold findings as activity and damage markers in dermatomyositis patients and their correlation with myositis antibodies. Arch Dermatol Res. 2023 Aug;315(6):1603-1613. doi: 10.1007/s00403-023-02554-0. Epub 2023 Feb 9. PMID: 36757439; PMCID: PMC9909127.


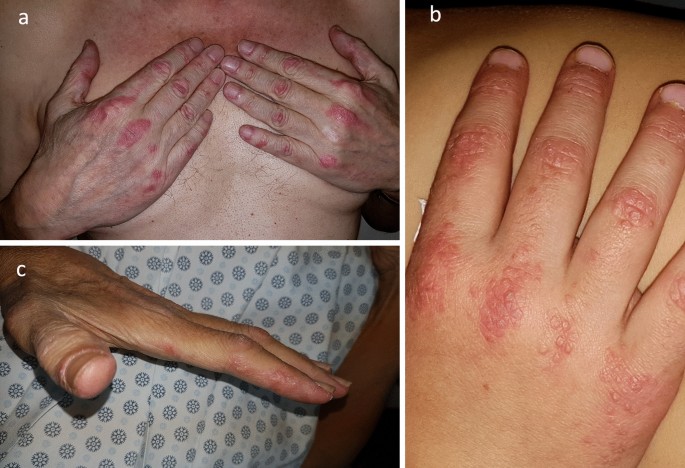


Score hands

Case 6: Ma R, Danko C. A Case Report on Dermatomyositis in a Female Patient with Facial Rash and Swelling. J Educ Teach Emerg Med. 2024 Oct 31;9(4):V1-V5. doi: 10.21980/J8506D. PMID: 39507471; PMCID: PMC11537729.


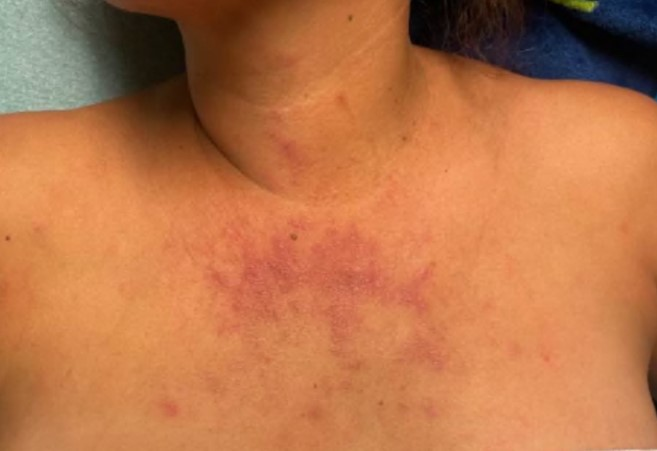


Score V-area neck (frontal)

# Case 7: Wang WY, Cheng ST. Sleeve Sign and Inverse Gottron's Papules in Anti-MDA5 Dermatomyositis. N Engl J Med. 2023 Sep 14;389(11):1032. doi: 10.1056/NEJMicm2301354. Epub 2023 Sep 9. PMID: 37694888.


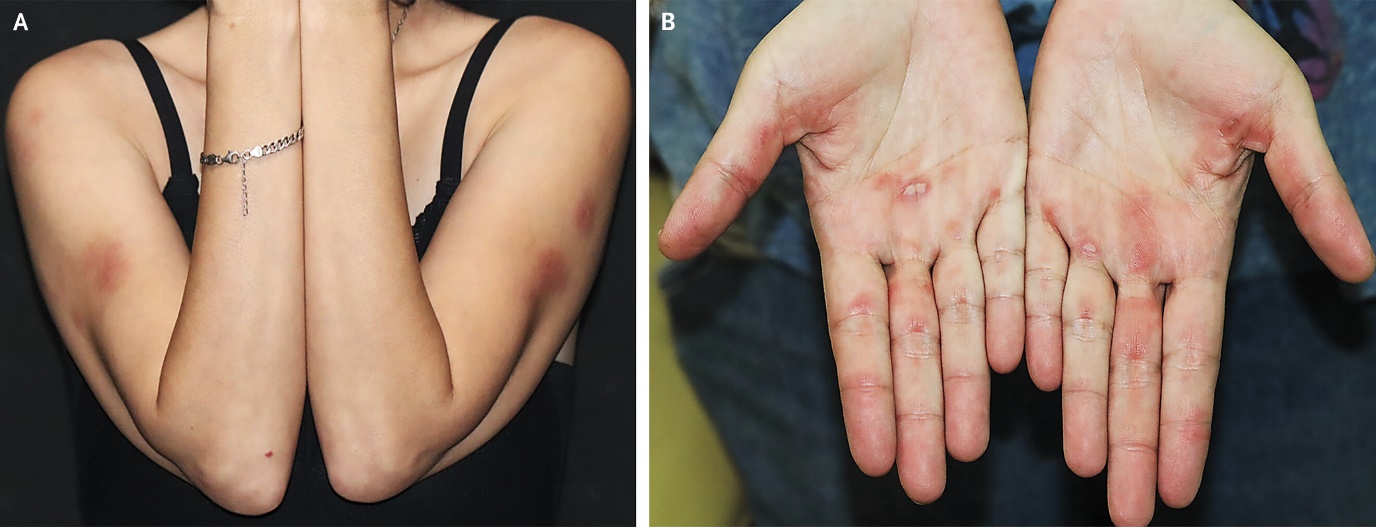


Score arm

Case 8: Gonzalez D, Gupta L, Murthy V, Gonzalez EB, Williamson KA, Makol A, Tan CL, Sulaiman FN, Shahril NS, Isa LM, Martín-Nares E, Aggarwal R. Anti-MDA5 dermatomyositis after COVID-19 vaccination: a case-based review. Rheumatol Int. 2022 Sep;42(9):1629-1641. doi: 10.1007/s00296-022-05149-6. Epub 2022 Jun 4. PMID: 35661906; PMCID: PMC9166182.


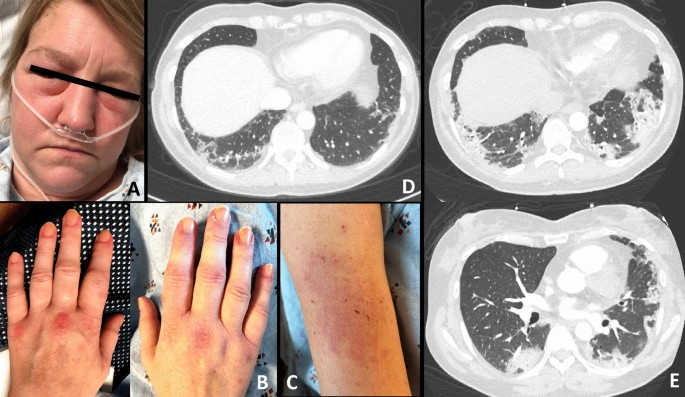


Score periorbital

Case 9: Gonzalez, D., Gupta, L., Murthy, V. *et al.* Anti-MDA5 dermatomyositis after COVID-19 vaccination: a case-based review. *Rheumatol Int* **42**, 1629–1641 (2022). https://doi.org/10.1007/s00296-022-05149-6


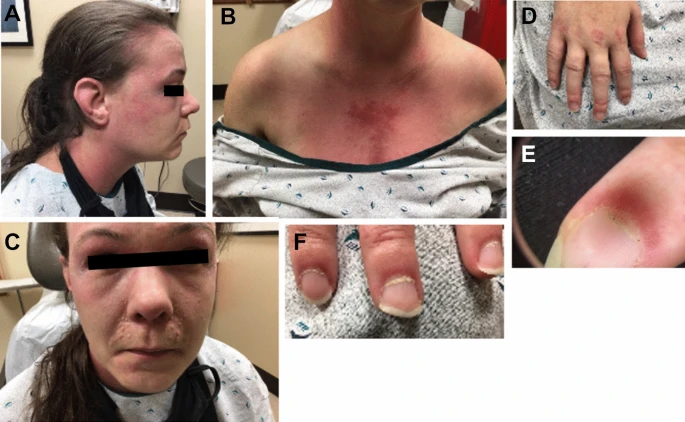


Score V-area neck (frontal)

Case 10: Gonzalez, D., Gupta, L., Murthy, V. *et al.* Anti-MDA5 dermatomyositis after COVID-19 vaccination: a case-based review. *Rheumatol Int* **42**, 1629–1641 (2022). https://doi.org/10.1007/s00296-022-05149-6


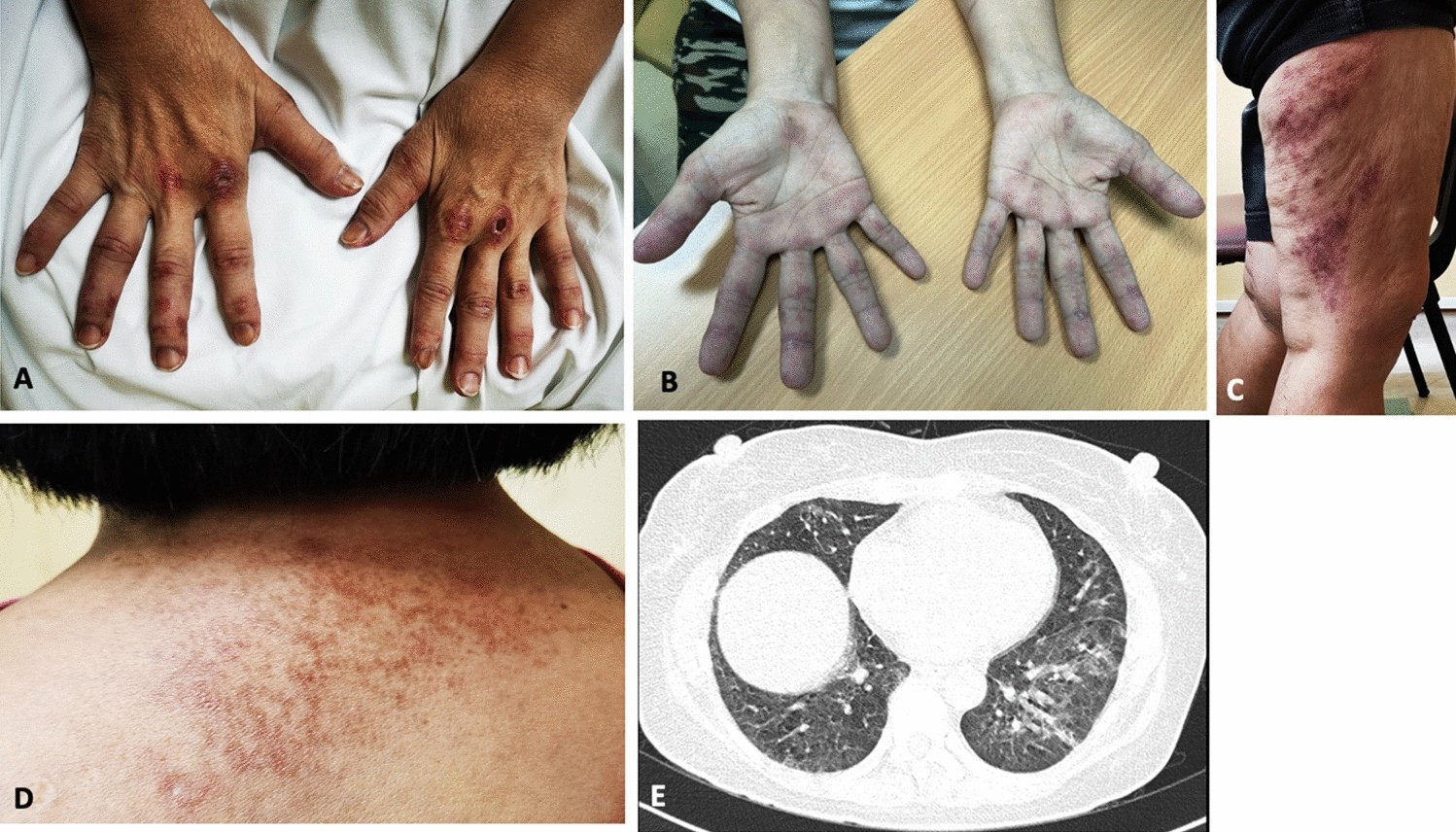


Score lateral upper thigh and hands

Case 11: Akagi T, Mukai T, Fujita S, Yamamoto T, Fukuda M, Morita Y. Severe oral stomatitis due to reactivation of herpes simplex virus type 1 in a methotrexate-treated patient with dermatomyositis. Immunol Med. 2021 Mar;44(1):56-60. doi: 10.1080/25785826.2020.1787585. Epub 2020 Jul 10. PMID: 32649847.


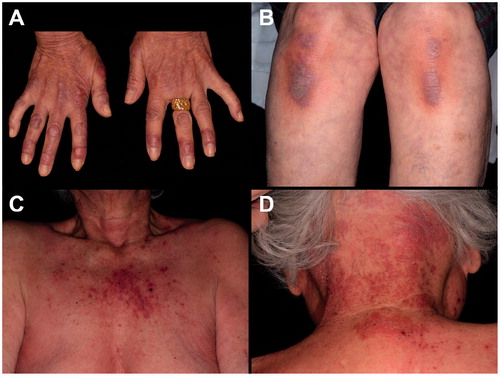


Score V-area neck (frontal)

Case 12: Connolly A, Gordon PA, Hannah J, Creamer D. The chameleon rash: a review of the polyphenotypic dermatoses of dermatomyositis. Clin Exp Dermatol. 2021 Aug;46(6):1016-1022. doi: 10.1111/ced.14689. Epub 2021 Jun 13. PMID: 33882159.


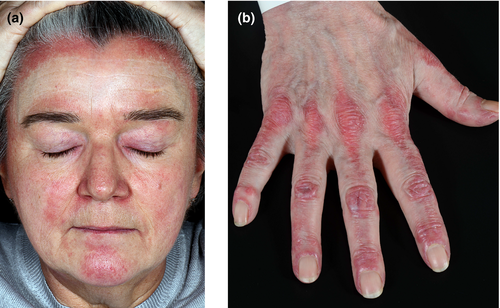


Score malar area and hand

Case 13: Suzuki A, Kondoh Y, Taniguchi H, Tabata K, Kimura T, Kataoka K, Ono K, Hashisako M, Fukuoka J. Lung histopathological pattern in a survivor with rapidly progressive interstitial lung disease and anti-melanoma differentiation-associated gene 5 antibody-positive clinically amyopathic dermatomyositis. Respir Med Case Rep. 2016 May 28;19:5-8. doi: 10.1016/j.rmcr.2016.05.008. PMID: 27354955; PMCID: PMC4910142.


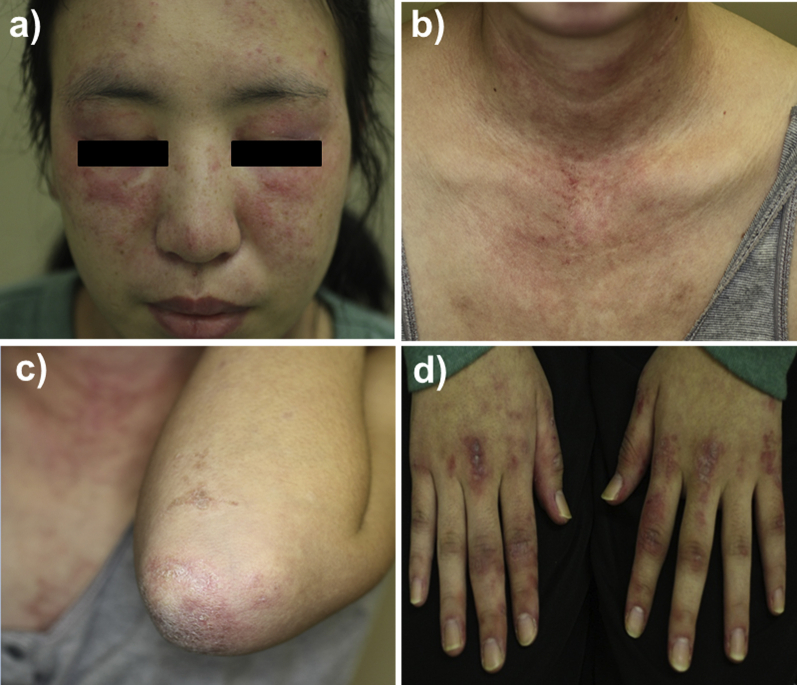


Score V-area neck (frontal)

Case 14: Lee SZ, Syed MT, Kumar P. Pneumomediastinum: A severe complication of dermatomyositis. Int J Case Rep Images 2021;12:101247Z01SL2021.


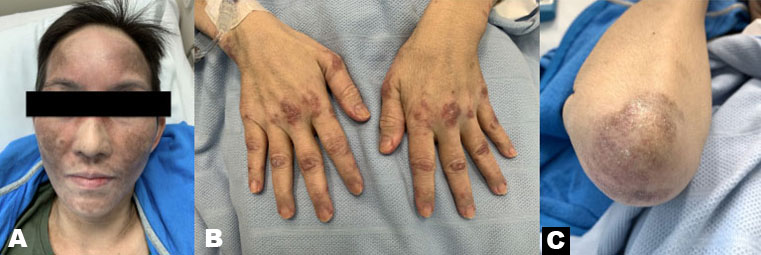


Score gottron’s not on hands and hands

Case 15: Kreuter, A., Lausch, S., Burmann, S.-.-N., Paschos, A. and Michalowitz, A.-.-L. (2022), Onset of amyopathic dermatomyositis following mRNA-based SARS-CoV-2 vaccination. J Eur Acad Dermatol Venereol, 36: e669-e672. <https://doi.org/10.1111/jdv.18211>


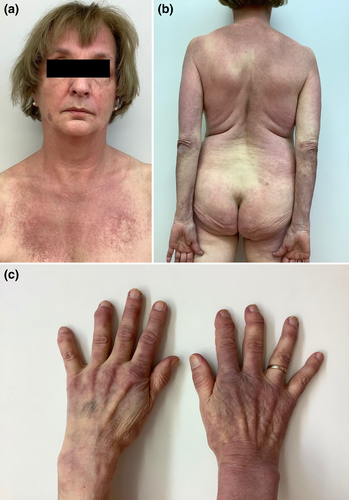

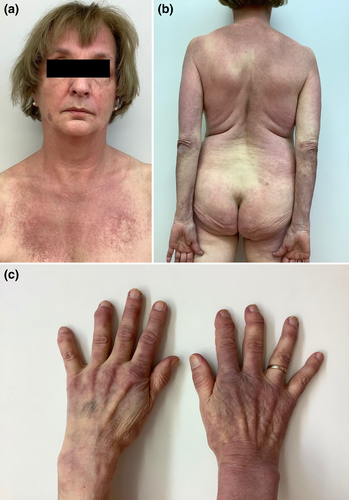


Score V-area neck (frontal) and hands

Case 16 Nguyen, M., Hai, J., Bovenberg, M. S, Wilken, R., Kiuru, M., Brassard, A., & Tartar, D. (2020). A patient with anti-NXP2-positive dermatomyositis and syphilis. *Dermatology Online Journal*, 26(2). http://dx.doi.org/10.5070/D3262047413 Retrieved from https://escholarship.org/uc/item/0783v3nx


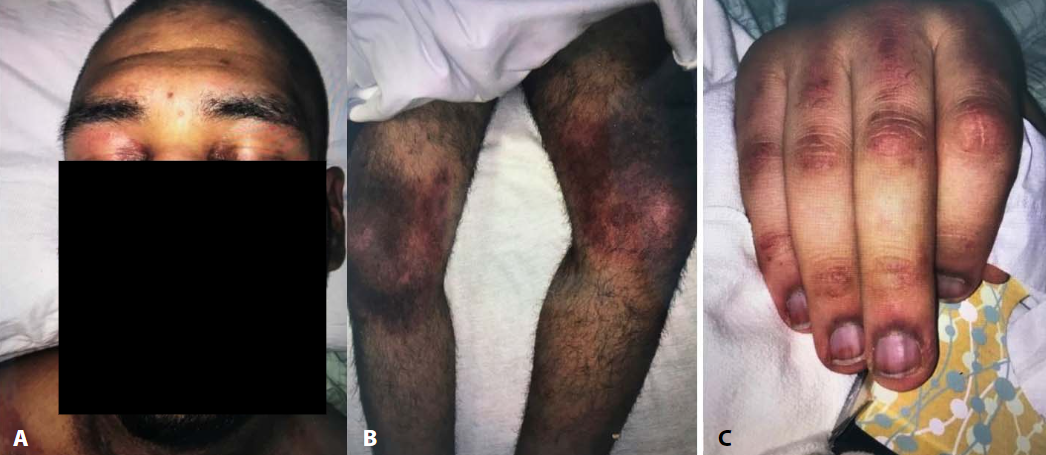


Score hand

Case 17: C. Cotter, E. Rudd, E. Williamson, M. Philippidou, A. Tewari, Anti‐Ku‐positive juvenile dermatomyositis, *Clinical and Experimental Dermatology*, Volume 47, Issue 2, 1 February 2022, Pages 425–427, <https://doi.org/10.1111/ced.14937>


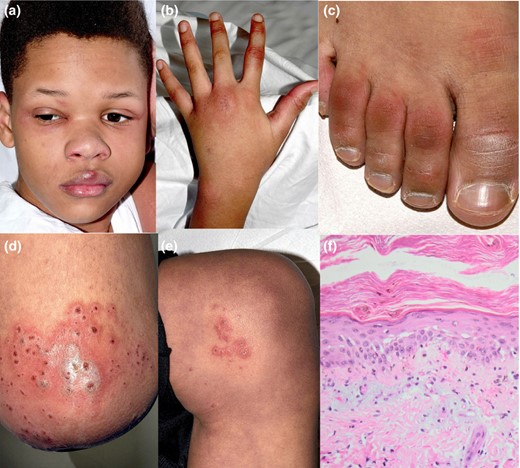


Score periorbital

Case 18: F. Galimberti, L. Kooistra, Y. Li, S. Chatterjee, A. P. Fernandez, Intravenous immunoglobulin is an effective treatment for refractory cutaneous dermatomyositis, *Clinical and Experimental Dermatology*, Volume 43, Issue 8, 1 December 2018, Pages 906–912, <https://doi.org/10.1111/ced.13607>


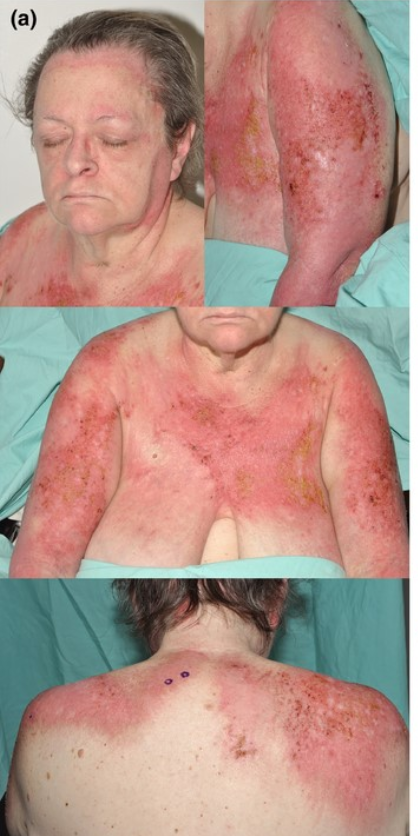


Score V-area neck (frontal)

Case 19 F. Galimberti, L. Kooistra, Y. Li, S. Chatterjee, A. P. Fernandez, Intravenous immunoglobulin is an effective treatment for refractory cutaneous dermatomyositis, *Clinical and Experimental Dermatology*, Volume 43, Issue 8, 1 December 2018, Pages 906–912, <https://doi.org/10.1111/ced.13607>


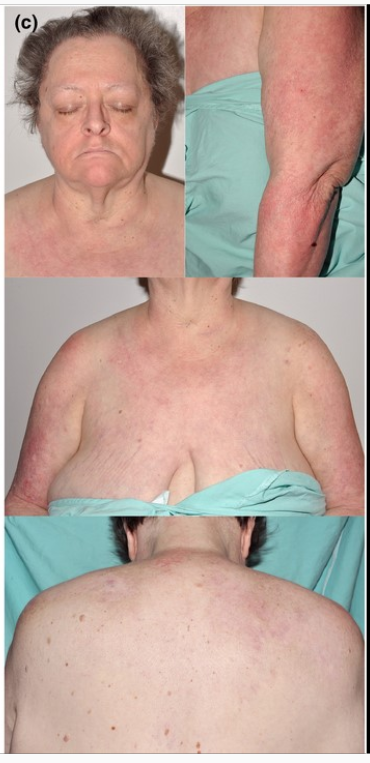


Score V-area neck (frontal)

Case 20: A. M. Molina‐Ruiz, F. Romero, L. Carrasco, F. Feltes, R. Haro, L. Requena, Amyophatic dermatomyositis presenting as a flagellated skin eruption with positive *MDA5* antibodies and thyroid cancer: a real association?, *Clinical and Experimental Dermatology*, Volume 40, Issue 8, 1 December 2015, Pages 887–890, <https://doi.org/10.1111/ced.12674>


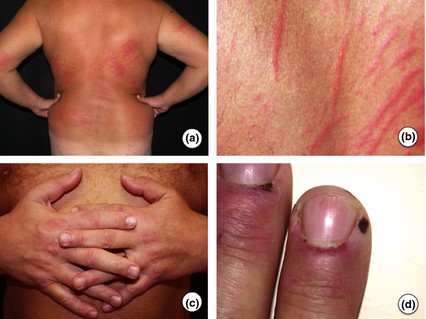


Score Shoulder

Case 21

Steininger, J., Günther, C. Aktuelle Aspekte zur Dermatomyositis. *Dermatologie* **75**, 153–162 (2024). https://doi.org/10.1007/s00105-023-05273-9


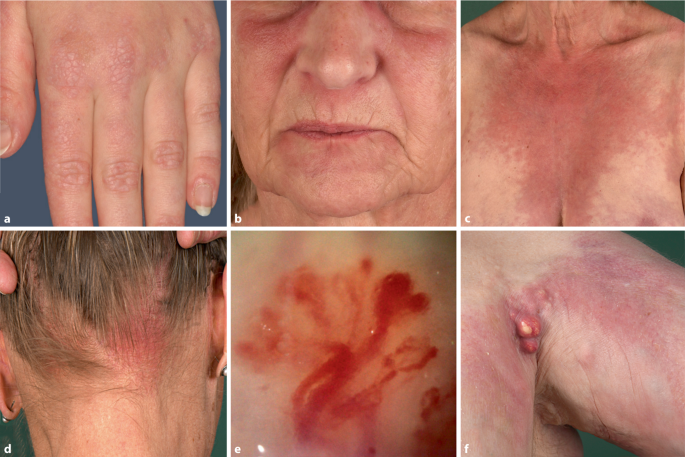


Score V-area neck (frontal)

Case 22: Barrón-Calvillo EE, García-Romero MT. Early-onset juvenile dermatomyositis: A report of two cases and review of the literature. *Pediatr Dermatol*. 2022; 39: 260–263. doi:[10.1111/pde.14930](https://doi.org/10.1111/pde.14930)


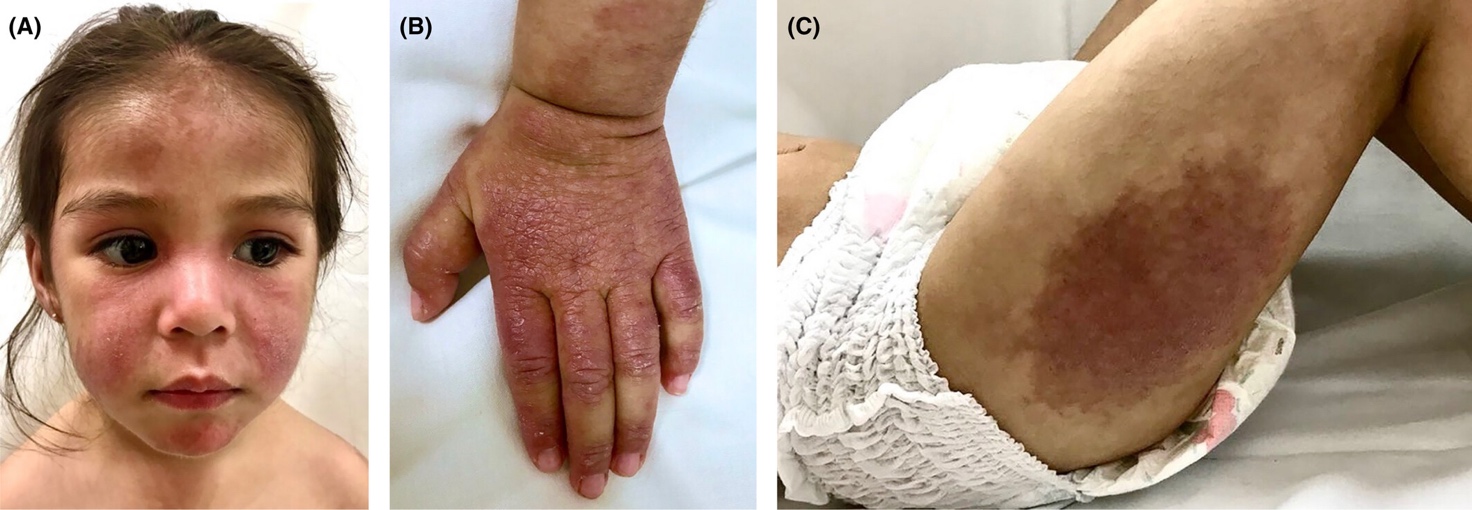


Score malar area

Case 23: ACR Image Competition (https://www.the-rheumatologist.org/article/acr-image-competition-2023-results-part-4/)


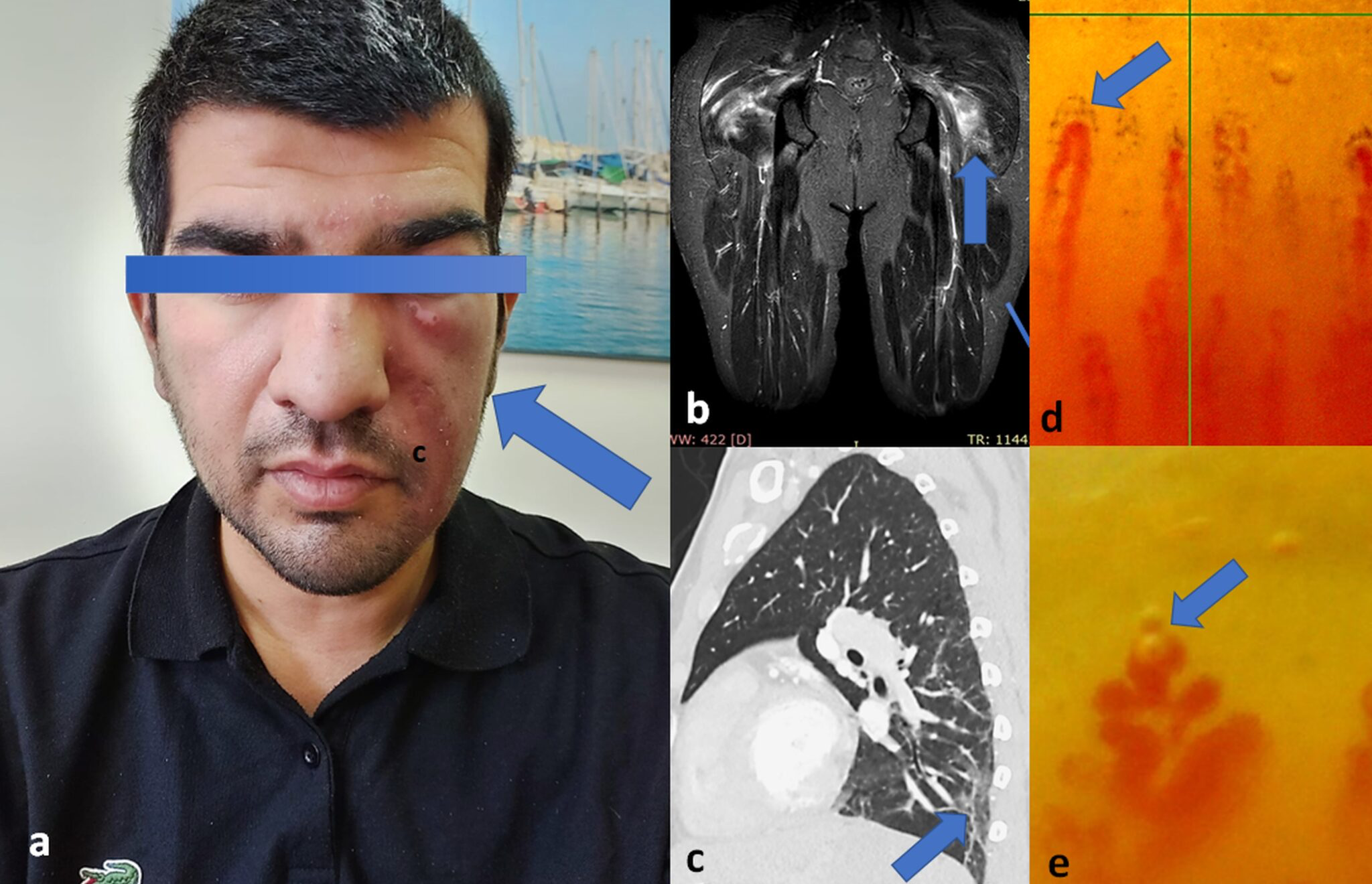


Score malar

Case 24: ACR Image Competition 2023 (https://www.the-rheumatologist.org/article/acr-image-competition-2023-results-part-5/)


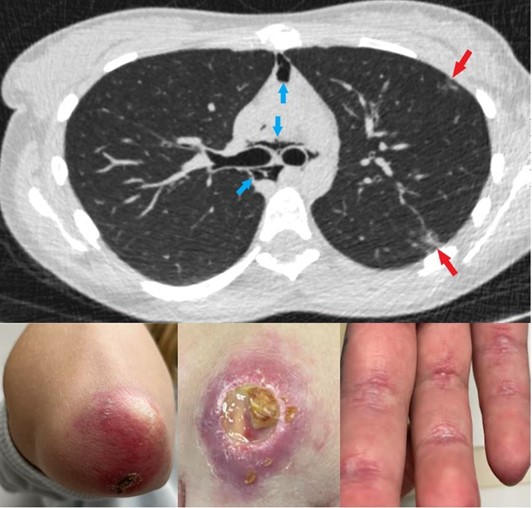


Score gottron’s not on hands

Case 25: ACR image competition (<https://www.the-rheumatologist.org/article/acr-image-competition-2023-results-part-3/>)


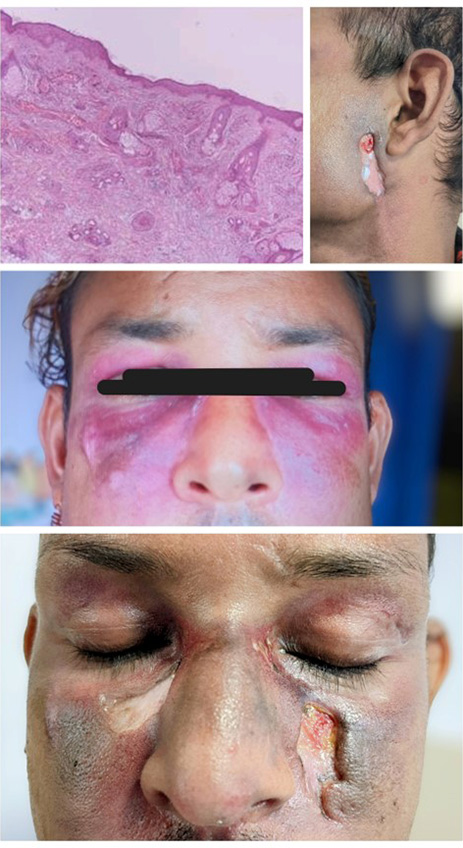


Score malar area

Case 26: ACR image competition (<https://www.the-rheumatologist.org/article/acr-image-competition-2023-results-part-3/>)


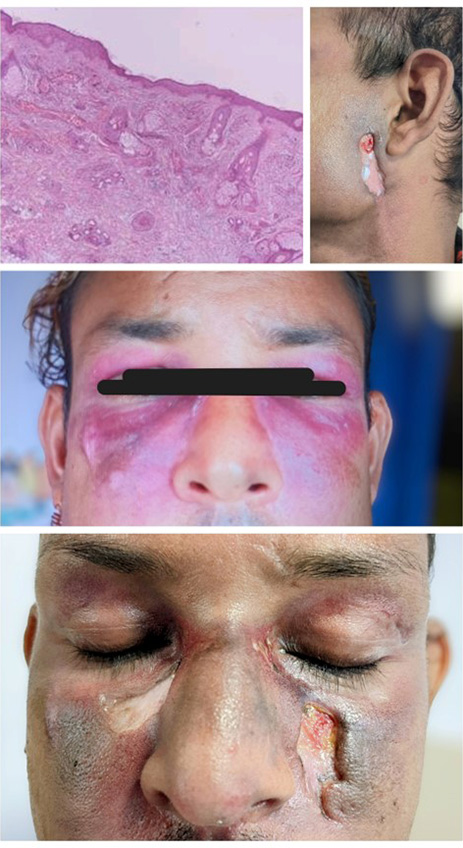


Score malar area

Case 27: ACR image competition (<https://www.the-rheumatologist.org/article/acr-image-competition-2023-results-part-1/>)


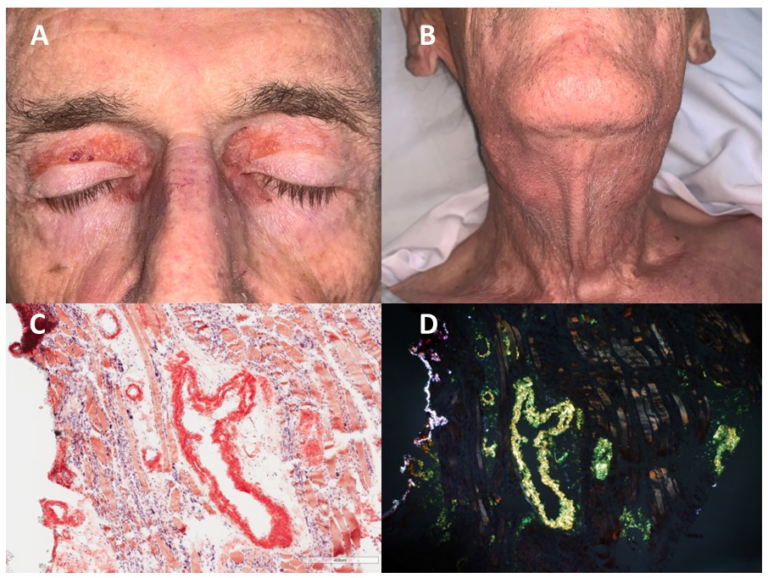


Score periorbital

Case 28: ACR Image Competition 5 (<https://www.the-rheumatologist.org/article/the-acr-image-competition-2021-results/>)


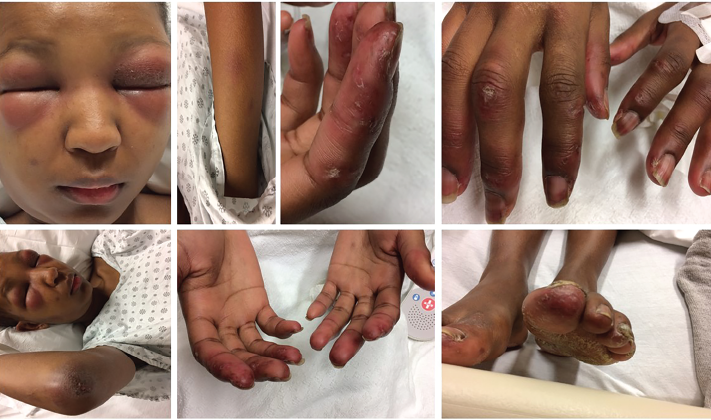


Score periorbital

Case 29: Chiu HH, Remalante PP, Nacianceno P, Velasco R, Larrazabal R, Zamora G.  Dermatomyositis Presenting as Life-threatening Hypercalcemia.  J Rheum Dis 2020;27:285-289.


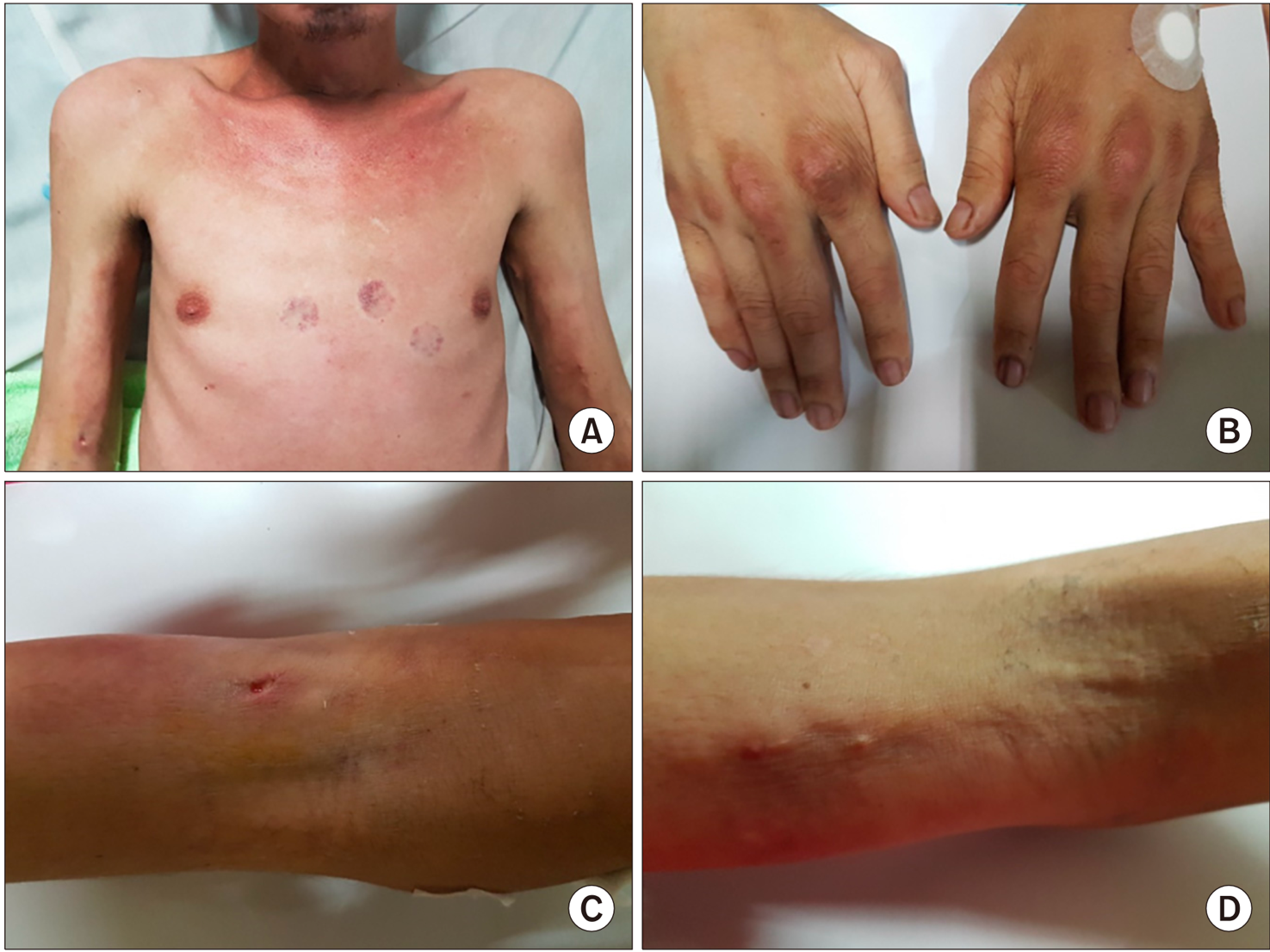


Score V-area neck (frontal)

Case 30: Amadore RA Jr, Bangayan RJ, Natividad TEL. A case of clinically amyopathic dermatomyositis in a Filipino woman. Int J Rheum Dis. 2022 Nov;25(11):1348-1352.


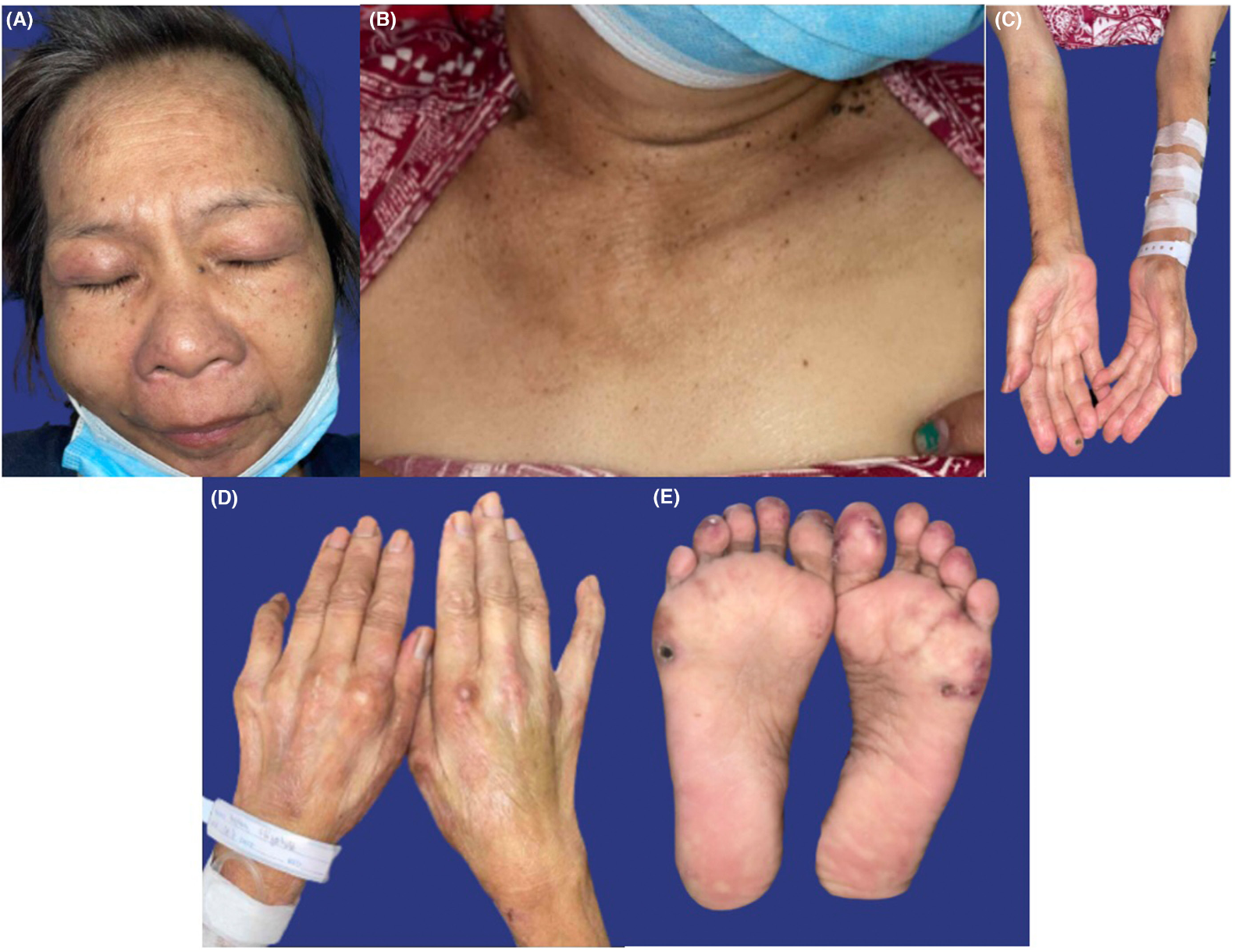


Score periorbital
